# Demographic, clinical, and immunological features in combined immunodeficiency patients: a comparative analysis of those with and without pulmonary manifestations – a multicenter study from Iran

**DOI:** 10.1186/s12890-026-04115-3

**Published:** 2026-01-31

**Authors:** Ghamartaj Khanbabaee, Matin Pourghasem, Mahnaz Jamee, Seyed Ahmad Tabatabaii, Mitra Khalili, Samin Sharafian, Mehrnaz Mesdaghi, Mahnaz Sadeghi-Shabestari, Armin Shirvani, Saeid Sadr, Arefeh Zahmatkesh, Samaneh Delavari, Narges Eslami, Nazanin Farahbakhsh, Mahboubeh Mansouri, Ebrahim Tabiei, Seyedeh Zalfa Modarresi, Abdolhamid Taghizadeh Behbahani, Golnaz Eslamian, Mazdak Fallahi, Javad Enayat, Shahrzad Fallah, Mahsa Pourghasem, Asghar Aghamohammadi, Zahra Chavoshzadeh

**Affiliations:** 1 Department of Pediatric Pulmonology, Mofid Children’s Hospital, Shahid Beheshti University of Medical Sciences, Tehran, Iran; 2 Pediatric Diseases Research Center, Guilan university of Medical Sciences, Rasht, Iran; 3 Pediatric Nephrology Research Center, Research Institute for Children’s Health, Shahid Beheshti University of Medical Sciences, Tehran, Iran; 4 Department of Radiology, Shahid Beheshti University of Medical Sciences, Tehran, Iran; 5 Immunology and Allergy Department, Mofid Children’s Hospital, Shahid Beheshti University of Medical Sciences, Tehran, Iran; 6 Immunology Research Center of Tabriz, TB and lung research center of Tabriz, children hospital, Tabriz University of medical science, Tabriz, Iran; 7 Department of Medical Education, Shahid Beheshti University of Medical Sciences, Tehran, Iran; 8 Research Center for Immunodeficiencies, Pediatrics Center of Excellence, Children’s Medical Center, Tehran University of Medical Sciences, Tehran, Iran; 9 Children Growth Disorder Research Center, Shahid Sadoughi University of Medical Sciences, Yazd, Iran; 10 Department of Pediatric Cardiology, Cardiovascular Research center, Rajaie cardiovascular institute, Tehran, Iran; 11 Shahid fahmideh children hospital, Iran University of medical sciences, Tehran, Iran; 12 Department of Pediatrics, Taleghani Children’s Hospital, Golestan University of Medical Sciences, Gorgan, Iran; 13 Department of Pharmacy, Pharmaceutical Sciences Branch (IAUPS), Islamic Azad University, Tehran, Iran; 14 Department of Pediatric Pulmonology, Akbar Children’s Hospital, Mashhad University of Medical Sciences, Mashhad, Iran

**Keywords:** Combined immunodeficiency, Primary immunodeficiency, Pulmonary manifestations, HRCT, Prognostic biomarkers, Bronchiectasis, Interstitial lung disease

## Abstract

**Background:**

Combined immunodeficiency (CID) involves profound defects in B and T lymphocyte development and function. This study examined clinical and immunological phenotypes of CID patients with and without pulmonary manifestations.

**Methods:**

This retrospective multicenter study included 53 CID patients diagnosed between 2009 and 2022 with available thoracic computed tomography scans. Patients were categorized based on pulmonary manifestations presence. Demographic, clinical, and laboratory characteristics were compared using conservative statistical thresholds (*P* < 0.01). All laboratory parameters were interpreted using age-adjusted pediatric reference ranges.

**Results:**

Among 53 patients (56.6% male), 43 had pulmonary abnormalities on HRCT. Common clinical features included skin lesions (43.4%), failure to thrive (34%), and autoimmunity (32.1%). HRCT revealed pneumonia (28.3%), bronchiectasis (18.9%), interstitial lung disease with BOOP-like pattern (3.8%), and other findings. Using age-adjusted pediatric reference ranges, profound immunological defects were confirmed: absolute lymphocyte count below the 5th percentile in 92% (49/53), CD3 + T cells below the 5th percentile in 94% (47/50 tested), CD4 + T cells below the 5th percentile in 96% (51/53), CD19 + B cells below the 5th percentile in 94% (50/53), and hypogammaglobulinaemia (IgG below the 5th percentile) in 98% (52/53). Patients with abnormal HRCT had significantly lower CD4 + T-cell counts (178 vs. 498 cells/µL; *P* = 0.008) and CD19 + B-cell counts (42 vs. 189 cells/µL; *P* = 0.009). Bronchoscopy identified Aspergillus fumigatus, Streptococcus pneumoniae, and multidrug-resistantAcinetobacter baumannii. Deceased patients showed significantly lower baseline platelets (183,000 vs. 266,000 cells/µL; *P* = 0.009), IgG (380 vs. 720 mg/dL; *P* = 0.007), and IgE (0.8 vs. 12 IU/mL; *P* = 0.008).

**Conclusion:**

Pulmonary manifestations affect 81.1% of Iranian CID patients. Low baseline platelets, IgG, and IgE constitute a robust prognostic triad for mortality (*P* = 0.009, *P* = 0.007, *P* = 0.008 respectively). Application of age-adjusted reference ranges revealed profound immunological defects. Systematic HRCT surveillance using low-dose protocols and distinguishing infectious sequelae from immune-mediated lung disease guides targeted management in resource-limited settings.

**Supplementary Information:**

The online version contains supplementary material available at 10.1186/s12890-026-04115-3.

## Introduction

Combined immunodeficiency (CID) encompasses a heterogeneous group of inborn errors of immunity characterized by significant impairments in B and T lymphocyte development and function. The reported incidence ranges from 1 in 5,000 to 1 in 100,000 live births across populations, but this probably underestimates the actual prevalence since many affected infants die before diagnosis [1–5]. In regions with high consanguinity rates such as Iran, autosomal recessive inheritance predominates, yielding comparatively higher disease burden [4–6].

Severe combined immunodeficiency (SCID), the most severe phenotype, typically manifests in early infancy with opportunistic infections, failure to thrive, chronic diarrhea, and dermatologic manifestations [7]. Although residual T-cell functionality in CID often produces milder presentations than classical SCID, underlying genetic defects affecting T-cell stimulatory function and B-cell isotype switching render patients vulnerable to bacterial and intracellular pathogens, particularly impacting pulmonary health [8–15]. Recent epidemiological studies confirm respiratory tract involvement as a predominant feature in primary immunodeficiency patients [10, 11].

Despite advances in molecular diagnostics, genetic characterization remains incomplete in many CID cohorts, including ours, which hampers personalized approaches such as gene therapy [12, 13, 16, 17].In the absence of routine genetic testing, the diagnosis of combined immunodeficiency continues to rely on widely accepted international clinical and immunological diagnostic criteria [18]. Pulmonary manifestations further compromise clinical outcomes, highlighting the importance of early recognition [15, 19].

Respiratory manifestations in CID include infectious processes (pneumonia, abscesses, empyema) and structural sequelae from recurrent infections, most notably bronchiectasis. Bronchiectasis represents irreversible airway changes that develop when repeated pneumonias damage the bronchial walls.

Once established, bronchiectatic airways harbor persistent bacterial colonization, perpetuating cycles of infection and inflammation [20–22]. In contrast, some patients develop interstitial lung disease patterns reflecting immune dysregulation rather than infection, such as bronchiolitis obliterans organizing pneumonia (BOOP) [20].

This difference in disease mechanisms affects treatment decisions. Bronchiectasis requires aggressive antimicrobial prophylaxis and airway clearance, while immune-mediated lung disease may necessitate immunomodulation. Immunologic profiling aids in identifying patients at increased risk, facilitating targeted surveillance [12].

Given the limited data on CID-associated pulmonary manifestations in Middle Eastern populations, this study retrospectively examined the phenotypic spectrum of CID patients with and without pulmonary involvement, integrating clinical, laboratory, and imaging data while applying age-adjusted pediatric reference ranges to accurately reflect the severity of immunological defects in this very young cohort [15, 19].

## Patients and methods

### Study population

This retrospective multicenter study involved 53 CID patients referred to Mofid Children’s Hospital and the Pediatric Center of Excellence (Shahid Beheshti University and Tehran University of Medical Sciences, Tehran, Iran) between 2009 and 2022. CID diagnosis was established by clinical immunologists using standardized European Society for Immunodeficiencies (ESID) and Pan-American Group for Immunodeficiency (PAGID) criteria [18], requiring evidence of combined T- and B-cell dysfunction with characteristic clinical features. Patients with secondary immunodeficiencies or isolated IEIs were excluded. Genetic testing was not routinely performed due to financial constraints in Iran during the study period (2009–2022).

### Data collection

Trained pediatric immunologists abstracted data from medical records using a structured 109-variable questionnaire (Supplementary File S1) organized into eleven domains: demographics, disease history, clinical presentation, infections, gastrointestinal/skin/autoimmune manifestations, organ involvement, treatments, imaging, and laboratory parameters.

Laboratory parameters—lymphocyte subsets (CD3+, CD4+, CD8+, CD19+) and immunoglobulin levels (IgG, IgA, IgM, IgE)—were measured during initial diagnostic evaluation prior to immunoglobulin replacement therapy (IgRT) initiation, ensuring values reflected inherent immunological defects rather than exogenous supplementation. Lymphocyte subset enumeration was performed using flow cytometry with age-appropriate reference ranges according to institutional protocols.

### Imaging and bronchoscopy

High-resolution computed tomography (HRCT) was performed for clinical indications (persistent cough, respiratory distress, recurrent pneumonia, abnormal auscultation) using low-dose protocols (effective dose ≤ 2 mSv) per ALARA principles. Median scan frequency was 2 per patient (range 1–4), reserved for clinical progression, treatment non-response, or pre-transplant evaluation.

Available scans were independently reviewed by a multidisciplinary team comprising pediatric pulmonologists with specialized expertise in immunodeficiency-related lung disease and board-certified radiologists. Discrepancies between reviewers were resolved through consensus discussion, integrating clinical context with radiological findings. Bronchoscopy with bronchoalveolar lavage (BAL) was performed selectively in four patients based on clinical judgment for suspected opportunistic infection or inadequate empirical therapy response.

Patients were categorized by pulmonary manifestations presence based on clinical evaluation and HRCT findings.

### Statistical analysis

Statistical analysis used SPSS version 26.0 (IBM Corp., Armonk, NY). Continuous variables were assessed for normality (Shapiro-Wilk test) and analyzed using independent t-tests or Mann-Whitney U tests. Categorical variables were compared using chi-square or Fisher’s exact tests when expected cell counts were below 5. Given the small sample size, particularly in the normal HRCT group (*n* = 10), statistical significance was conservatively defined as *P* < 0.01 rather than *P* < 0.05 to reduce type I error risk. All tests were two-tailed.

### Ethical approval

This study received Ethics Committee approval from Shahid Beheshti University of Medical Sciences (IR.SBMU.MSP.REC.1399.692). All procedures conformed to institutional standards and the 1964 Helsinki Declaration. Informed consent was waived given the retrospective nature and use of de-identified data.

## Results

### Demographic data

The study included 53 CID patients: 30 males (56.6%) and 23 females (43.4%). Based on HRCT findings, 43 patients (81.1%) had pulmonary abnormalities (abnormal HRCT group: 23 males, 20 females) and 10 (18.9%) had normal findings (normal HRCT group: 7 males, 3 females) (*P* = 0.498).

Parental consanguinity was documented in 38 patients (71.7%). Family history of primary immunodeficiency or unexplained infant deaths was reported in 10 cases (18.9%). At data collection, 43 patients (81.1%) were alive and 10 (18.9%) deceased. All deceased patients were from the abnormal HRCT group.

Median age at disease onset was 10 months (IQR: 2.3–18), median age at diagnosis was 18 months (IQR: 7-50.4), and median diagnostic delay was 2 months (IQR: 0-12.4). Patients with pulmonary manifestations tended toward earlier symptom onset, though not statistically significant (*P* = 0.671). Table 1 summarizes demographic characteristics.

**Table 1:** Demographic characteristics of CID patients

Parameter	Total (*n* = 53)	Abnormal HRCT (*n* = 43)	Normal HRCT (*n* = 10)	*P*-value
Age at onset (months), median (IQR)	10 (2.3–18)	7 (1-16.5)	14 (3–20)	0.618
Age at diagnosis (months), median (IQR)	18 (7-50.4)	13 (6–42)	36 (10–84)	0.652
Diagnostic delay (months), median (IQR)	2 (0-12.4)	3.5 (0.5–18)	1 (0-4.5)	0.730
Male sex, n (%)	30 (56.6)	23 (53.5)	7 (70.0)	0.498
Parental consanguinity, n (%)	38 (71.7)	32 (74.4)	6 (60.0)	0.442
Family history of PID, n (%)	10 (18.9)	10 (23.3)	0 (0)	0.178
Mortality, n (%)	10 (18.9)	10 (23.3)	0 (0)	0.183

*Abbreviations: CID* Combined immunodeficiency, *HRCT* High-resolution computed tomography, *IQR* Interquartile range, *PID* Primary immunodeficiency disease

Data are medians (IQR) for continuous variables and numbers (percentages) for categorical variables

*P-*values from Mann-Whitney U test (continuous) and Fisher's exact test (categorical)

*P*<0.01 considered significant

### Clinical presentations

Skin lesions were the most common non-infectious manifestation (*n* = 23, 43.4%), including eczema, cellulitis, and warts, with similar prevalence between groups (41.9% vs. 50.0%, *P* = 0.730). Failure to thrive occurred in 18 patients (34%), more frequently with pulmonary manifestations (37.2% vs. 20.0%), though not statistically significant (*P* = 0.464). Hepatomegaly (20.9%) and splenomegaly (25.6%) were observed exclusively in the abnormal HRCT group, typically associated with systemic infections such as sepsis or disseminated BCGosis.

Autoimmune manifestations were documented in 17 patients (32.1%): cytopenias (*n* = 6), JIA-like arthritis (*n* = 2), IBD-like enteropathy (*n* = 2), thyroiditis (*n* = 1), alopecia (*n* = 3), and vasculitis (*n* = 3). Table 2 summarizes clinical manifestations.

**Table 2:** Clinical manifestations in CID patients

Clinical Feature	Total (*n* = 53)	Abnormal HRCT (*n* = 43)	Normal HRCT (*n* = 10)	*P*-value
Failure to thrive, n (%)	18 (34.0)	16 (37.2)	2 (20.0)	0.464
Skin lesions, n (%)	23 (43.4)	18 (41.9)	5 (50.0)	0.730
Autoimmunity, n (%)	17 (32.1)	13 (30.2)	4 (40.0)	0.709
Hepatomegaly, n (%)	9 (17.0)	9 (20.9)	0 (0)	0.327
Splenomegaly, n (%)	11 (20.8)	11 (25.6)	0 (0)	0.198
Lymphadenopathy, n (%)	7 (13.2)	7 (16.3)	0 (0)	0.323
Oral candidiasis, n (%)	10 (18.9)	9 (20.9)	1 (10.0)	0.665
BCGosis, n (%)	18 (34.0)	18 (41.9)	0 (0)	0.052
Cardiac involvement, n (%)	5 (9.4)	5 (11.6)	0 (0)	0.570
PNS involvement, n (%)	8 (15.1)	5 (11.6)	3 (30.0)	0.163
Malignancy, n (%)	2 (3.8)	2 (4.7)	0 (0)	1.000

*Abbreviations: PNS* Paranasal sinuses, *BCG*, Bacille Calmette-Guérin

*P-*values from Fisher's exact test

*P*<0.01 considered significant

### Imaging and Microbiological findings

Thoracic CT scans were performed for all patients. Systematic multidisciplinary review revealed pulmonary abnormalities in 43 patients (81.1%):

- Pneumonia with consolidation or ground-glass opacities: 15 patients (28.3%).
- Bronchiectasis: 10 patients (18.9%).
- Interstitial lung disease with BOOP-like pattern: 2 patients (3.8%).
- Atelectasis: 2 patients (3.8%).
- Pulmonary nodules: 1 patient (1.9%).
- Pleural complications (empyema, effusion): 1 patient (1.9%).

The two patients with interstitial lung disease presented with BOOP-like patterns characterized by diffuse ground-glass opacities and mosaic attenuation (supplementary Figures S1.1 and S1.2), suggesting immune dysregulation rather than active infection. Of the 43 patients with abnormal HRCT, 41 had evidence of infectious or post-infectious manifestations (pneumonia, bronchiectasis, or pathogen-positive BAL), while 2 exhibited ILD with BOOP-like pattern suggestive of immune dysregulation. Subgroup comparison of mortality between infectious and non-infectious pulmonary manifestations was not performed due to the small sample size in the non-infectious group (*n* = 2).

BAL was performed in four patients. Microbiological analysis identified Aspergillus fumigatus (two patients), Streptococcus pneumoniae (one patient), and multidrug-resistant Acinetobacterbaumannii (one patient).

### Laboratory findings

Application of age-adjusted pediatric reference ranges revealed profound immunological defects in the majority of patients:

- Absolute lymphocyte count below the 5th percentile for age: 92% (49/53).
- CD3 + T cells below the 5th percentile: 94% (47/50 tested).
- CD4 + T cells below the 5th percentile: 96% (51/53).
- CD19 + B cells below the 5th percentile: 94% (50/53).
- Hypogammaglobulinaemia (IgG below the 5th percentile for age): 98% (52/53).
- IgA below the 5th percentile: 95% (50/53).
- IgM below the 5th percentile: 73% (39/53).
- IgE below the 5th percentile: 100% (53/53).

Laboratory comparisons revealed significant differences in lymphocyte subsets between groups. Patients with abnormal HRCT demonstrated significantly lower CD4 + T-cell counts (median 178 vs. 498 cells/µL; *P* = 0.008) and CD19 + B-cell counts (median 42 vs. 189 cells/µL; *P* = 0.009) compared to those with normal HRCT, further supporting the association between profound adaptive immune defects and pulmonary manifestations.

Median WBC (8,550 vs. 7,400 cells/µL, *P* = 0.622) and lymphocyte counts (3,384 vs. 2,520 cells/µL, *P* = 0.194) were higher in the pulmonary manifestation group, though not reaching statistical significance at *P* < 0.01. CD8 + T-cell counts (median 112 vs. 210 cells/µL, *P* = 0.087) were lower with pulmonary manifestations but did not reach statistical significance. Median IgG was lower with pulmonary manifestations (765 vs. 999 mg/dL, *P* = 0.330), while IgA (147 vs. 91 mg/dL, *P* = 0.503) and IgM (127 vs. 82 mg/dL, *P* = 0.321) were higher.

Deceased patients exhibited significantly lower baseline values constituting a prognostic triad: platelets (183,000 vs. 266,000 cells/µL; *P* = 0.009), IgG (380 vs. 720 mg/dL; *P* = 0.007), and IgE (0.8 vs. 12 IU/mL; *P* = 0.008). These parameters remained significant under the stringent *P* < 0.01 threshold. Table 3 summarizes laboratory data.

**Table 3:** Laboratory parameters in CID patients

Parameter	Abnormal HRCT (*n* = 43)	Normal HRCT (*n* = 10)	*P*-value	Deceased (*n* = 10)	Survivors (*n* = 43)	*P*-value
WBC (×10³/µL)	8.6 (5.2–12.1)	7.4 (4.8–10.2)	0.622	6.2 (3.1–11.4)	7.8 (4.2–14.5)	0.214
Lymphocytes (cells/µL)	3,384 (1,820-5,240)	2,520 (1,450-4,100)	0.194	1,120 (320-2,800)	1,850 (680-4,200)	0.089
Hemoglobin (g/dL)	10.1 (8.2–11.9)	12.1 (9.8–13.4)	0.053	9.8 (7.2–11.5)	10.5 (8.1–13.2)	0.312
Platelets (×10³/µL)	266 (198–342)	278 (220–365)	0.890	183 (110–250)	266 (180–420)	0.009
IgG (mg/dL)	765 (420-1,080)	999 (680-1,320)	0.330	380 (180–720)	720 (320-1,200)	0.007
IgA (mg/dL)	147 (58–245)	91 (35–178)	0.503	22 (0–58)	35 (0–92)	0.156
IgM (mg/dL)	127 (65–198)	82 (42–152)	0.321	38 (12–110)	52 (18–145)	0.221
IgE (IU/mL)	13 (4–28)	19 (8–35)	0.514	0.8 (0-2.1)	12 (2–45)	0.008
CD3+ (cells/µL)	312 (120–680)	689 (280-1,450)	0.012	480 (120-1,200)	720 (180-1,800)	0.134
CD4+ (cells/µL)	178 (65–420)	498 (180–980)	0.008	280 (60–680)	420 (110-1,100)	0.198
CD8+ (cells/µL)	112 (40–280)	210 (85–520)	0.087	180 (50–520)	260 (80–720)	0.267
CD19+ (cells/µL)	42 (10–120)	189 (65–420)	0.009	80 (0-220)	145 (0-480)	0.112

*Abbreviations: WBC* White blood cells, *Ig* Immunoglobulin, *CD* Cluster of differentiation

All laboratory parameters were interpreted using age-adjusted pediatric reference ranges according to institutional standards and ESID/PAGID diagnostic criteria [18]. Values below the age-specific 5th percentile were considered abnormal. All immunological parameters obtained pre-IgRT. CD3+ was measured in 50 patients, CD4+, CD8+, and CD19+ in 53 patients. NK cell enumeration was performed in only 10 patients (18.9%), all showing markedly reduced counts, precluding meaningful statistical comparison

Values are medians (IQR)

*P*-values from Mann-Whitney U test

Bold indicates P<0.01.

## Discussion

This retrospective multicenter study examined CID patients with and without pulmonary manifestations in a Middle Eastern cohort characterized by high consanguinity (71.7%). The respiratory system is frequently the predominant affected organ in IEIs, with pulmonary infections often representing initial presentation [23]. The high prevalence of pulmonary abnormalities (81.1%) highlights respiratory manifestations as a significant concern requiring systematic surveillance [19, 24], consistent with French prospective data showing respiratory infections as the leading hospitalization cause in primary immunodeficiency [25].

When we applied age-adjusted pediatric reference ranges, we found profound immunological defects: absolute lymphocyte counts were below the 5th percentile in 92% of patients, CD3 + T cells in 94%, CD4 + T cells in 96%, CD19 + B cells in 94%, and hypogammaglobulinaemia was present in 98%.This underscores the importance of age-appropriate reference ranges in pediatric immunodeficiency evaluation, particularly in CID where the majority present in infancy.

Patients with abnormal HRCT demonstrated significantly lower CD4 + T-cell counts (178 vs. 498 cells/µL; *P* = 0.008) and CD19 + B-cell counts (42 vs. 189 cells/µL; *P* = 0.009) compared to those with normal HRCT. These findings indicate that more profound T-cell and B-cell deficiency associates with increased pulmonary manifestation risk, supporting the role of both cellular and humoral immunity in protecting against respiratory infections. In resource-limited settings where comprehensive genetic testing is often unavailable, flow cytometry-based lymphocyte enumeration can provide quantitative immunophenotyping. This approach enables confirmation of combined T- and B-cell deficiency, risk stratification for pulmonary manifestations, and helps guide HSCT timing decisions.

We most commonly found pneumonia (28.3%) and bronchiectasis (18.9%). Bronchiectasis represents chronic structural damage from recurrent infections, developing through repeated pneumonias causing chronic inflammation and progressive bronchial wall destruction (supplementary Figure S1.3). Once established, bronchiectatic airways harbor persistent bacterial colonization, perpetuating infection-inflammation cycles [20–22]. Two patients (3.8%) exhibited interstitial lung disease with BOOP-like patterns, suggesting immune dysregulation as the primary driver rather than active infection. This phenotype resembles patterns in other primary immunodeficiencies such as CVID [20]. However, due to the limited number of patients with non-infectious pulmonary manifestations (*n* = 2 with ILD/BOOP-like pattern), a comparative analysis of mortality or immunological differences between infectious and non-infectious subgroups was not statistically feasible.

This difference in disease mechanisms affects treatment decisions. Bronchiectasis requires intensified antimicrobial prophylaxis and aggressive exacerbation treatment, while ILD patterns reflecting immune dysregulation may necessitate immunomodulatory approaches. These findings align with USIDNET registry data highlighting respiratory manifestations’ impact on CID outcomes [14] and regional studies confirming high respiratory infection prevalence in primary immunodeficiency [6, 26].

Distinguishing infectious from immune-mediated pulmonary injury increasingly relies on integrated clinical, imaging, and mechanistic approaches. Experimental immunodeficiency models have provided valuable insights into the pathophysiologic pathways driving these divergent phenotypes and support translational interpretation of clinical lung manifestations in CID [27]. Advanced imaging studies have further characterized the radiologic spectrum of pulmonary disease across primary immunodeficiencies, highlighting patterns such as bronchiectasis, ILD, nodular disease, and BOOP-like changes that assist in distinguishing chronic infection from dysregulated inflammation [28]. These radiologic findings correlate closely with clinical outcomes, reinforcing the need for systematic surveillance and multidisciplinary management, particularly in cases of bronchiectasis where aggressive antimicrobial prophylaxis and structured airway-clearance strategies are essential [29].

HRCT served as the primary pulmonary evaluation modality in this study [21, 30, 31]. We employed low-dose protocols with median 2 scans per patient (range 1–4), reserving repeat imaging for clinical deterioration, inadequate treatment response, or pre-transplant evaluation.

Early CID diagnosis is essential since severe phenotypes require prompt intervention and delayed diagnosis significantly compromises prognosis. Machine learning models for patient data analysis represent promising strategies for facilitating earlier diagnosis in under-resourced regions [32]. Our study revealed patients with pulmonary manifestations were diagnosed earlier (median 13 vs. 36 months) but experienced longer diagnostic delays (3.5 vs. 1 month). This paradox reflects differences in symptom onset and severity. Patients with pulmonary manifestations presented with early, severe respiratory symptoms within the first year, prompting earlier attention but encountering resource-limited challenges including limited specialized testing access. Conversely, patients without pulmonary manifestations had later, milder symptom onset, resulting in later but more rapid diagnosis once symptoms appeared. These findings align with Arabian Peninsula cohort data suggesting high consanguinity and diagnostic challenges contribute to delayed interventions [15], supported by studies emphasizing early IEI diagnosis importance [10, 33].

All deceased patients were from the abnormal HRCT group, supporting the association between respiratory manifestations and mortality, though the difference did not reach statistical significance (*P* = 0.183) due to small normal HRCT group size (*n* = 10). This is consistent with literature indicating respiratory infections as main morbidity and mortality causes in predominantly antibody deficiency [34], aligning with USIDNET registry data [14].

A particularly significant finding was the prognostic triad identified in deceased patients: significantly lower baseline platelets (183,000 vs. 266,000 cells/µL; *P* = 0.009), IgG (380 vs. 720 mg/dL; *P* = 0.007), and IgE (0.8 vs. 12 IU/mL; *P* = 0.008)—all measured pre-IgRT. All three parameters remained significant at the stringent *P* < 0.01 threshold, suggesting they are robust mortality predictors. Thrombocytopenia may reflect bone marrow involvement, chronic inflammation, consumptive coagulopathy, or autoimmune thrombocytopenia—all recognized CID manifestations [9]. Hypogammaglobulinemia indicates severe humoral compromise, evident through poor vaccine responses and recurrent infections despite quantitatively adequate IgG. Profoundly low IgE suggests severe B-cell class switching and maturation impairment, requiring intact T-cell help and CD40-CD40L signaling. This contrasts with Hyper-IgE syndromes where elevated IgE is characteristic. This prognostic triad warrants validation in larger prospective cohorts and may guide early risk stratification and HSCT decision-making.

The significant reduction in CD4 + T-cell and CD19 + B-cell counts in patients with pulmonary manifestations (*P* = 0.008 and *P* = 0.009 respectively) indicates that profound deficiency in both adaptive immune compartments contributes to respiratory infection susceptibility. This pattern has been observed in other immunodeficiencies. HIV patients with lower CD4 + counts experience community-acquired respiratory infections more frequently [35]. In CVID, bronchiectasis associates with decreased CD4 + counts [36], and more profound T-cell defects link to higher respiratory infection rates compared to X-linked agammaglobulinemia [37]. Our findings extend these observations to CID, demonstrating that both T-cell and B-cell deficiency severity correlate with pulmonary manifestation risk. Such variability in T- and B-cell impairment is consistent with the increasingly recognized complexity and evolving clinical spectrum of inborn errors of immunity [38].

Bronchiectasis, identified in 18.9% of pulmonary involvement patients, represents a well-established IEI manifestation. Its presence at diagnosis portends poor prognosis. In a 900-patient bronchiectasis cohort, IEI was the underlying cause in 16% [22]. These findings emphasize infection control’s critical importance for preventing irreversible lung injury in immunocompromised patients. Studies demonstrate appropriate antimicrobial therapy may decelerate progression and alter the trajectory toward bronchiectasis. Early treatment reduces chronic lung disease risk and attenuates infection severity, particularly opportunistic ones. Investigators advocate aggressive infection control and early intervention given persistent infections and immune dysregulation in CID [39–42].

Systematic reviews emphasize CID presentation heterogeneity, highlighting genetic characterization necessity for predicting pulmonary and systemic outcomes [13, 43]. Comprehensive genetic data were unavailable in our cohort due to testing costs and limited availability during 2009–2022 [38]. Although gene sequencing is preferred for early diagnosis and genotype-phenotype correlations [44], CID diagnoses in our setting were established using ESID and PAGID criteria incorporating clinical phenotype, lymphocyte enumeration, and functional assessments [18]. This constraint limited insights into specific molecular defects underlying pulmonary manifestations, emphasizing accessible genetic testing need in resource-limited settings and improved newborn screening programs, as recommended by international guidelines [12, 13, 19].

This study’s novelty lies in several aspects: one of the largest systematic pulmonary manifestation evaluations in a Middle Eastern CID cohort with high consanguinity (71.7%); identification of a robust prognostic triad (low platelets *P* = 0.009, low IgG *P* = 0.007, low IgE *P* = 0.008) significant under stringent *P* < 0.01 threshold; demonstration that lower CD4+ (*P* = 0.008) and CD19+ (*P* = 0.009) counts associate with pulmonary manifestations; systematic HRCT characterization using low-dose protocols with multidisciplinary review; application of age-adjusted pediatric reference ranges revealing profound immunological defects (92–98% abnormalities); insights into diagnostic patterns and delays in resource-limited settings informing clinical practice in similar populations worldwide.

Study limitations include retrospective design introducing potential selection bias toward severely affected patients, small sample size particularly in the normal HRCT group (*n* = 10) limiting statistical power for some comparisons, absence of comprehensive genetic data precluding genotype-phenotype correlations, lack of functional B-cell assays such as antigen-specific antibody responses limiting humoral immunity assessment beyond quantitative immunoglobulin levels, selective bronchoscopy in only four patients potentially underestimating opportunistic infection burden, NK cell enumeration in only 10 patients (18.9%) precluding meaningful analysis, and lack of longitudinal pulmonary function testing and serial imaging data. Despite limitations, this study represents one of the largest Middle Eastern CID cohorts with systematic pulmonary evaluation, offering insights into disease expression in high-consanguinity contexts generalizable to similar resource-limited settings.

## Conclusion

This study demonstrates high pulmonary manifestation prevalence (81.1%) in Iranian CID children, with substantial parental consanguinity (71.7%). Pneumonia and bronchiectasis predominate as infectious manifestations and chronic infectious sequelae, respectively, while interstitial lung disease with BOOP-like patterns reflects immune dysregulation. A novel prognostic triad—baseline thrombocytopenia, hypogammaglobulinemia, and hypo-IgE-emia—emerged as robust mortality predictors (*P* = 0.009, *P* = 0.007, *P* = 0.008 respectively, all meeting the stringent *P* < 0.01 threshold), identifying high-risk patients who may benefit from early HSCT evaluation.

Application of age-adjusted pediatric reference ranges revealed profound immunological defects in the vast majority: 92% lymphopenia, 94% CD3 + T-cell deficiency (measured in 50 patients), 96% CD4 + T-cell deficiency, 94% CD19 + B-cell deficiency, and 98% hypogammaglobulinaemia. Patients with pulmonary manifestations had significantly lower CD4 + T-cell counts (*P* = 0.008) and CD19 + B-cell counts (*P* = 0.009). This underscores the critical importance of both adaptive immune compartments in protecting against respiratory infections.These findings emphasize the necessity of using age-appropriate reference ranges when evaluating pediatric immunodeficiency patients.

Timely recognition and routine monitoring through low-dose HRCT and comprehensive laboratory assessments are essential for detecting early respiratory involvement. Distinguishing infectious sequelae from immune-mediated lung disease guides targeted management. Optimizing early diagnosis through improved newborn screening, facilitating genetic testing access, and implementing personalized interventions may reduce disease burden and improve quality of life. Our findings align with international consensus on CID management and highlight systematic pulmonary surveillance importance in this vulnerable population [45].

## Supplementary Information

Supplementary Material 1. Structured Data Collection Questionnaire - A comprehensive 109-variable questionnaire across 11 domains used for systematic data collection in this study.

Supplementary Material 2. Figure S1.1 - HRCT demonstrating an extensive BOOP-like pattern in a 2-year-old male CID patient, characterized by diffuse bilateral ground-glass opacities and mosaic attenuation, illustrating immune-mediated interstitial lung disease. Figure S1.2 - HRCT demonstrating a BOOP-like pattern in a 5-year-old male CID patient, showing bilateral ground-glass opacities with mosaic attenuation, characteristic of immune dysregulation-mediated lung injury. Figure S1.3 - HRCT in a 12-year-old male with CID demonstrating cylindrical bronchiectasis, bronchial wall thickening, and a 'tree-in-bud' pattern, representing chronic infectious sequelae of recurrent pneumonia.

## Data Availability

Datasets are not publicly available due to patient privacy but can be obtained from the corresponding author upon reasonable request.

## References

[1] Nourizadeh M, Borte S, Fazlollahi MR, Hammarström L, Pourpak Z. A new IL-2RG gene mutation in an X-linked SCID identified through TREC/KREC screening: a case report. Iran J Allergy Asthma Immunol. 2015;14(4):457–61. 26547715

[2] Abolhassani H, Cheraghi T, Rezaei N, Aghamohammadi A, Hammarström L. Common variable immunodeficiency or Late-Onset combined immunodeficiency: A new hypomorphic JAK3 patient and review of the literature. J Investig Allergol Clin Immunol. 2015;25(3):218–20. 26182690

[3] Al-Herz W, Notarangelo LD, Sadek A, Buckley R, USIDNET Consortium. Combined immunodeficiency in the united States and kuwait: comparison of patients’ characteristics and molecular diagnosis. Clin Immunol. 2015;161(2):170–3. 26248333 10.1016/j.clim.2015.07.013 PMC4869855

[4] Lee PPW, Chan KW, Chen TX, Jiang LP, Wang XC, Zeng HS, et al. Molecular diagnosis of severe combined immunodeficiency–identification of IL2RG, JAK3, IL7R, DCLRE1C, RAG1, and RAG2 mutations in a cohort of Chinese and Southeast Asian children. J Clin Immunol. 2011;31(2):281–96. 21184155 10.1007/s10875-010-9489-z

[5] Yee A, De Ravin SS, Elliott E, Ziegler JB. Contributors to the Australian paediatric surveillance Unit. Severe combined immunodeficiency: a National surveillance study. Pediatr Allergy Immunol. 2008;19(4):298–302. 10.1111/j.1399-3038.2007.00646.x 18221464

[6] Mohammadzadeh I, Moazzami B, Ghaffari J, Aghamohammadi A, Rezaei N. Primary immunodeficiency diseases in Northern Iran. Allergol Immunopathol (Madr). 2017;45(3):244–50. 28237128 10.1016/j.aller.2016.11.001

[7] Bonilla FA, Khan DA, Ballas ZK, Chinen J, Frank MM, Hsu JT, et al. Practice parameter for the diagnosis and management of primary immunodeficiency. J Allergy Clin Immunol. 2015;136(5):1186–e12051. 26371839 10.1016/j.jaci.2015.04.049

[8] Bousfiha A, Jeddane L, Picard C, Al-Herz W, Ailal F, Chatila T, et al. Human inborn errors of immunity: 2019 update of the IUIS phenotypical classification. J Clin Immunol. 2020;40(1):66–81. 32048120 10.1007/s10875-020-00758-x PMC7082388

[9] Tangye SG, Al-Herz W, Bousfiha A, Chatila T, Cunningham-Rundles C, Etzioni A, et al. Human inborn errors of immunity: 2019 update on the classification from the international union of immunological societies expert committee. J Clin Immunol. 2020;40(1):24–64. 31953710 10.1007/s10875-019-00737-x PMC7082301

[10] Esenboga S, Oguz B, Cagdas D, Karaatmaca B, Emiralioglu N, Yalcin E, et al. Respiratory system findings in pediatric patients with primary immunodeficiency. Pediatr Pulmonol. 2021;56(12):4011–9. 34499824 10.1002/ppul.25657

[11] Kim VHD, Upton JEM, Derfalvi B, Hildebrand KJ, McCusker C. Inborn errors of immunity (primary immunodeficiencies). Allergy Asthma Clin Immunol. 2025;20(Suppl 3):76. 39780212 10.1186/s13223-024-00938-z PMC11714877

[12] Al-Herz W, Ziyab AH, Adeli M, Al Farsi T, Al-Hammadi S, Al Kuwaiti AA, et al. Epidemiology of combined immunodeficiencies affecting cellular and humoral immunity- a multicentric retrospective cohort study from the Arabian Peninsula. Clin Immunol. 2023;254:109696. 37481010 10.1016/j.clim.2023.109696

[13] Redmond MT, Scherzer R, Prince BT. Novel genetic discoveries in primary immunodeficiency disorders. Clin Rev Allergy Immunol. 2022;63(1):55–74. 35020168 10.1007/s12016-021-08881-2 PMC8753955

[14] Durkee-Shock J, Zhang A, Liang H, Wright H, Magnusson J, Garabedian E, et al. Morbidity, Mortality, and therapeutics in combined immunodeficiency: data from the USIDNET registry. J Allergy Clin Immunol Pract. 2022;10(5):1334–e13416.35172220 10.1016/j.jaip.2022.01.042

[15] Owayed A, Al-Herz W. Sinopulmonary complications in subjects with primary immunodeficiency. Respir Care. 2016;61(8):1067–72. 26957648 10.4187/respcare.04479

[16] Fischer A, Hacein-Bey-Abina S, Cavazzana-Calvo M. Gene therapy of primary T cell immunodeficiencies. Gene. 2013;525(2):170–3. 23583799 10.1016/j.gene.2013.03.092

[17] Afzal SY, MacDougall MS, McGhee SA. Immunodeficiency. Gene therapy for primary immune deficiency. Allergy Asthma Proc. 2024;45(5):384-8. 39294901 10.2500/aap.2024.45.240054

[18] Dvorak CC, Haddad E, Heimall J, Dunn E, Buckley RH, Kohn DB, et al. The diagnosis of severe combined immunodeficiency (SCID): the primary immune deficiency treatment consortium (PIDTC) 2022 definitions. J Allergy Clin Immunol. 2023;151(2):539–46. 36456361 10.1016/j.jaci.2022.10.022 PMC9905311

[19] Abolhassani H, Chou J, Bainter W, Platt CD, Tavassoli M, Momen T, et al. Clinical, immunologic, and genetic spectrum of 696 patients with combined immunodeficiency. J Allergy Clin Immunol. 2018;141(4):1450–8. 28916186 10.1016/j.jaci.2017.06.049

[20] Khanbabaee G, Khazaii F, Chavoshzadeh Z, Rekabi M, Ghomi Z, Zeinali V, et al. Interstitial lung diseases (ILD) in common variable immunodeficiency (CVID) patients: a study from Iran. BMC Immunol. 2024;16(1):45. 10.1186/s12865-024-00640-0 PMC11251223 39014337

[21] Jesenak M, Banovcin P, Jesenakova B, Babusikova E. Pulmonary manifestations of primary immunodeficiency disorders in children. Front Pediatr. 2014;2:77. 25121077 10.3389/fped.2014.00077 PMC4110629

[22] Brower KS, Del Vecchio MT, Aronoff SC. The etiologies of non-CF bronchiectasis in childhood: a systematic review of 989 subjects. BMC Pediatr. 2014;14:4. 25492164 10.1186/s12887-014-0299-y PMC4275950

[23] Patrawala M, Cui Y, Peng L, Fuleihan RL, Garabedian EK, Patel K, et al. Pulmonary disease burden in primary immune deficiency disorders: data from USIDNET registry. J Clin Immunol. 2020;40(2):340–9. 31919711 10.1007/s10875-019-00738-w

[24] Thalhammer J, Kindle G, Nieters A, Rusch S, Seppänen MRJ, Fischer A, et al. Initial presenting manifestations in 16,486 patients with inborn errors of immunity include infections and noninfectious manifestations. J Allergy Clin Immunol. 2021;148(5):1332–e13415. 33895260 10.1016/j.jaci.2021.04.015

[25] Coignard-Biehler H, Mahlaoui N, Pilmis B, Barlogis V, Brosselin P, De Vergnes N, et al. A 1-Year prospective French nationwide study of emergency hospital admissions in children and adults with primary immunodeficiency. J Clin Immunol. 2019;39(7):702–12. 31401750 10.1007/s10875-019-00658-9

[26] Eissa H, Thakar MS, Shah AJ, Logan BR, Griffith LM, Dong H, et al. Posttransplantation late complications increase over time for patients with SCID: A primary immune deficiency treatment consortium (PIDTC) landmark study. J Allergy Clin Immunol. 2024;153(1):287–96. 37793572 10.1016/j.jaci.2023.09.027 PMC11294800

[27] Basheer F, Sertori R, Liongue C, Ward AC. Zebrafish: A relevant genetic model for human primary immunodeficiency (PID) disorders? Int J Mol Sci. 2023;24(7):6468. 37047441 10.3390/ijms24076468 PMC10095346

[28] Grenier PA, Brun AL, Longchampt E, Lipski M, Mellot F, Catherinot E. Primary immunodeficiency diseases of adults: a review of pulmonary complication imaging findings. Eur Radiol. 2024;34(6):4142–54. 37935849 10.1007/s00330-023-10334-7 PMC11166740

[29] Wall LA, Wisner EL, Gipson KS, Sorensen RU. Bronchiectasis in primary antibody deficiencies: A multidisciplinary approach. Front Immunol. 2020;11:522. 32296433 10.3389/fimmu.2020.00522 PMC7138103

[30] Feydy A, Sibilia J, De Kerviler E, Zagdanski AM, Chevret S, Fermand JP, et al. Chest high resolution CT in adults with primary humoral immunodeficiency. Br J Radiol. 1996;69(828):1108–16. 9135465 10.1259/0007-1285-69-828-1108

[31] Owens C. Radiology of diffuse interstitial pulmonary disease in children. Eur Radiol. 2004;14(Suppl 4):L2–12. 14752574 10.1007/s00330-003-2037-y

[32] Mayampurath A, Ajith A, Anderson-Smits C, Chang SC, Brouwer E, Johnson J, et al. Early diagnosis of primary immunodeficiency disease using clinical data and machine learning. J Allergy Clin Immunol Pract. 2022;10(11):3002–e30075. 36108921 10.1016/j.jaip.2022.08.041

[33] Gutierrez MJ, Nino G, Sun D, Restrepo-Gualteros S, Sadreameli SC, Fiorino EK, et al. The lung in inborn errors of immunity: from clinical disease patterns to molecular pathogenesis. J Allergy Clin Immunol. 2022;150(6):1314–24. 36244852 10.1016/j.jaci.2022.08.024 PMC9826631

[34] Cinetto F, Scarpa R, Rattazzi M, Agostini C. The broad spectrum of lung diseases in primary antibody deficiencies. Eur Respir Rev. 2018;27(149):180019. 30158276 10.1183/16000617.0019-2018 PMC9488739

[35] Lamas CC, Coelho LE, Grinsztejn BJ, Veloso VG. Community-acquired lower respiratory tract infections in HIV-infected patients on antiretroviral therapy: predictors in a contemporary cohort study. Infection. 2017;45(6):801–9. 28660356 10.1007/s15010-017-1041-0 PMC5873951

[36] Maglione PJ, Overbey JR, Radigan L, Bagiella E, Cunningham-Rundles C. Pulmonary radiologic findings in common variable immunodeficiency: clinical and immunological correlations. Ann Allergy Asthma Immunol. 2014;113(4):452–9. 24880814 10.1016/j.anai.2014.04.024 PMC4177442

[37] Weinberger T, Fuleihan R, Cunningham-Rundles C, Maglione PJ. Factors beyond lack of antibody govern pulmonary complications in primary antibody deficiency. J Clin Immunol. 2019;39(4):440–7. 31089938 10.1007/s10875-019-00640-5 PMC6939305

[38] Walter JE, Ziegler JB, Ballow M, Cunningham-Rundles C. Advances and challenges of the decade: the Ever-Changing clinical and genetic landscape of immunodeficiency. J Allergy Clin Immunol Pract. 2023;11(1):107–15. 36610755 10.1016/j.jaip.2022.11.007

[39] Haidopoulou K, Calder A, Jones A, Jaffe A, Sonnappa S. Bronchiectasis secondary to primary immunodeficiency in children: longitudinal changes in structure and function. Pediatr Pulmonol. 2009;44(7):669–75. 19514055 10.1002/ppul.21036

[40] Quartier P, Debré M, De Blic J, de Sauverzac R, Sayegh N, Jabado N, et al. Early and prolonged intravenous Immunoglobulin replacement therapy in childhood agammaglobulinemia: a retrospective survey of 31 patients. J Pediatr. 1999;134(5):589–96. 10228295 10.1016/s0022-3476(99)70246-5

[41] Tarzi MD, Grigoriadou S, Carr SB, Kuitert LM, Longhurst HJ. Clinical immunology review series: an approach to the management of pulmonary disease in primary antibody deficiency. Clin Exp Immunol. 2009;155(2):147–55. 19128358 10.1111/j.1365-2249.2008.03851.x PMC2675244

[42] Winkelstein JA, Marino MC, Lederman HM, Jones SM, Sullivan K, Burks AW, et al. X-linked agammaglobulinemia: report on a united States registry of 201 patients. Med (Baltim). 2006;85(4):193–202. 10.1097/01.md.0000229482.27398.ad 16862044

[43] IJspeert H, Edwards ESJ, O’Hehir RE, Dalm VASH, van Zelm MC. Update on inborn errors of immunity. J Allergy Clin Immunol. 2025;155(3):740–51. 39724969 10.1016/j.jaci.2024.12.1075

[44] Zeng Y, Ying W, Wang W, Hou J, Liu L, Sun B, et al. Clinical and genetic characteristics of BCG disease in Chinese children: a retrospective study. J Clin Immunol. 2023;43(4):756–68. 36662455 10.1007/s10875-022-01422-2

[45] Rivers L, Gaspar HB. Severe combined immunodeficiency: recent developments and guidance on clinical management. Arch Dis Child. 2015;100(7):667–72. 25564533 10.1136/archdischild-2014-306425

