# Supplementary material for: Demographic, clinical, and immunological features in combined immunodeficiency patients: a comparative analysis of those with and without pulmonary manifestations – a multicenter study from Iran
Source: BMC Pulm Med. 2026 Jan 31;26:100. doi: 10.1186/s12890-026-04115-3 (PMC12947537; doi:10.1186/s12890-026-04115-3)
Supplement: Supplementary file 1 — Supplementary Material 1. Structured Data Collection Questionnaire - A comprehensive 109-variable questionnaire across 11 domains used for systematic data collection in this study. [file 12890_2026_4115_MOESM1_ESM.docx]

**Supplementary File S1**

**Structured Data Collection Questionnaire for Combined Immunodeficiency (CID) Patients**

**Study Title:** Demographic, Clinical, and Immunological Features in Combined Immunodeficiency Patients: A Comparative Analysis of Those With and Without Pulmonary Complications

**Institution:** Mofid Children's Hospital and Pediatric Center of Excellence, Tehran, Iran

**Study Period:** 2009-2022

**Ethics Approval:** IR.SBMU.MSP.REC.1399.692

**Total Variables:** 109 across 11 domains

**SECTION 1: DEMOGRAPHICS (7 Variables)**

| **#** | **Variable** | **Data Type** | **Coding/ Options** |
| --- | --- | --- | --- |
| 1 | Patient ID | Numeric | Unique identifier |
| 2 | Sex | Categorical | 1=Male, 2=Female |
| 3 | Age at Evaluation | Numeric | Months |
| 4 | City of Residence | Text | Free text |
| 5 | Ethnicity | Categorical | 1=Persian, 2=Turkmen, 3=Lor, 4=Kurd, 5=Afghan, 6=Other |
| 6 | Parental Consanguinity | Binary | 1=Yes, 0=No |
| 7 | Family History of PID | Binary | 1=Yes, 0=No |

**SECTION 2: DISEASE HISTORY (6 Variables)**

| **#** | **Variable** | **Data Type** | **Coding/ Options** |
| --- | --- | --- | --- |
| 8 | Age at Onset of Symptoms | Numeric | Months |
| 9 | Age at Diagnosis | Numeric | Months |
| 10 | Diagnostic Delay | Calculated | Age at Diagnosis - Age at Onset (months) |
| 11 | Vital Status | Binary | 1=Alive, 0=Deceased |
| 12 | Age at Death | Numeric | Months (if deceased) |
| 13 | Cause of Death | Text | Free text (if deceased) |

**SECTION 3: FIRST CLINICAL PRESENTATION (3 Variables)**

| **#** | **Variable** | **Data Type** | **Coding/ Options** |
| --- | --- | --- | --- |
| 14 | First Clinical Presentation | Categorical | 1=Respiratory infection, 2=Chronic diarrhea, 3=Failure to thrive, 4=Skin manifestation, 5=BCGosis, 6=Other |
| 15 | First Presentation Category | Categorical | 1=Respiratory, 2=Gastrointestinal, 3=Growth, 4=Skin, 5=Vaccine-related, 6=Other |
| 16 | Primary Diagnosis at Presentation | Text | Free text |

**SECTION 4: INFECTIOUS DISORDERS (15 Variables)**

| **#** | **Variable** | **Data Type** | **Coding/ Options** |
| --- | --- | --- | --- |
| 17 | Age at First Infection | Numeric | Months |
| 18 | Pneumonia | Binary | 1=Yes, 0=No |
| 19 | Sinusitis | Binary | 1=Yes, 0=No |
| 20 | Bronchiectasis | Binary | 1=Yes, 0=No |
| 21 | ENT Infections | Binary | 1=Yes, 0=No |
| 22 | Meningitis | Binary | 1=Yes, 0=No |
| 23 | Abscess | Binary | 1=Yes, 0=No |
| 24 | Peritonitis | Binary | 1=Yes, 0=No |
| 25 | Urinary Tract Infection | Binary | 1=Yes, 0=No |
| 26 | Sepsis | Binary | 1=Yes, 0=No |
| 27 | Osteomyelitis | Binary | 1=Yes, 0=No |
| 28 | Chronic Diarrhea | Binary | 1=Yes, 0=No |
| 29 | Candidiasis | Binary | 1=Yes, 0=No |
| 30 | BCGosis | Binary | 1=Yes, 0=No |
| 31 | Other Infections | Text | Specify |

**Note on Variable 20 (Bronchiectasis):**
Bronchiectasis in this study represents chronic structural damage resulting from recurrent bacterial infections, distinct from the ILD patterns described in the imaging section below. This is post-infectious pathology characterized by irreversible bronchial wall destruction from repeated pneumonias.

**SECTION 5: GASTROINTESTINAL MANIFESTATIONS (4 Variables)**

| **#** | **Variable** | **Data Type** | **Coding/ Options** |
| --- | --- | --- | --- |
| 32 | Age at First GI Symptom | Numeric | Months |
| 33 | Enteropathy | Binary | 1=Yes, 0=No |
| 34 | Enteropathy Type | Categorical | 1=IBD-like, 2=Other |
| 35 | Failure to Thrive (FTT) | Binary | 1=Yes (weight <3rd percentile), 0=No |

**SECTION 6: SKIN MANIFESTATIONS (5 Variables)**

| **#** | **Variable** | **Data Type** | **Coding/ Options** |
| --- | --- | --- | --- |
| 36 | Age at First Skin Manifestation | Numeric | Months |
| 37 | Skin Disease | Binary | 1=Yes, 0=No |
| 38 | Skin Disease Type | Categorical | 1=Eczema, 2=Warts, 3=Cellulitis, 4=Other |
| 39 | Current Skin Status | Categorical | 1=Complete remission, 2=Relapse, 3=Active disease |
| 40 | Cellulitis | Binary | 1=Yes, 0=No |

**SECTION 7: AUTOIMMUNITY AND ATOPY (6 Variables)**

| **#** | **Variable** | **Data Type** | **Coding/ Options** |
| --- | --- | --- | --- |
| 41 | Age at First Autoimmune Manifestation | Numeric | Months |
| 42 | Autoimmunity | Binary | 1=Yes, 0=No |
| 43 | Autoimmunity Type | Categorical | 1=Cytopenia (AIHA/ITP/neutropenia), 2=Arthritis (JIA-like), 3=Vasculitis, 4=Thyroiditis, 5=Alopecia, 6=Other |
| 44 | Age at First Atopic Manifestation | Numeric | Months |
| 45 | Atopy | Binary | 1=Yes, 0=No |
| 46 | Atopic Disorders | Categorical | 1=Eczema, 2=Food allergy, 3=Asthma, 4=Other |

**SECTION 8: OTHER ORGAN SYSTEMS (35 Variables)**

This section includes comprehensive assessment of involvement across multiple organ systems. Variables 47-81 cover:

• Malignancy (type, timing)

• Granulomatous disease

• Rheumatologic manifestations

• Hematologic abnormalities beyond cytopenias

• Cardiovascular involvement

• Hepatobiliary disease (hepatomegaly, splenomegaly, liver function abnormalities)

• Endocrine dysfunction

• Neurologic manifestations

• Renal involvement

• Lymphoproliferative disorders

• Other systemic manifestations

*Note: Full detailed list available in complete study dataset. Abbreviated here for brevity. Key variables include hepatomegaly, splenomegaly, lymphadenopathy, cardiac involvement, and malignancy which are specifically analyzed in the manuscript.*

**SECTION 9: TREATMENT AND MANAGEMENT (11 Variables)**

| **#** | **Variable** | **Data Type** | **Coding/ Options** |
| --- | --- | --- | --- |
| 82 | Corticosteroid Therapy | Binary | 1=Yes, 0=No |
| 83 | Chemotherapy/Immunosuppressants | Binary | 1=Yes, 0=No |
| 84 | Type of Immunosuppressant | Text | Specify (if applicable) |
| 85 | Immunoglobulin Replacement Therapy | Categorical | 0=No, 1=Low-dose, 2=High-dose |
| 86 | Antibiotic Prophylaxis | Binary | 1=Yes, 0=No |
| 87 | Antiviral Prophylaxis | Binary | 1=Yes, 0=No |
| 88 | Antifungal Prophylaxis | Binary | 1=Yes, 0=No |
| 89 | HSCT Candidate | Binary | 1=Yes, 0=No |
| 90 | Surgical Interventions | Binary | 1=Yes, 0=No |
| 91 | Other Treatments | Text | Specify |

**SECTION 10: IMAGING FINDINGS (5 Variables)**

**High-Resolution Computed Tomography (HRCT) of Chest**

| **#** | **Variable** | **Data Type** | **Coding/ Options** |
| --- | --- | --- | --- |
| 92 | HRCT Performed | Binary | 1=Yes, 0=No |
| 93 | HRCT Findings | Multi-select | See HRCT coding table below |
| 94 | Other HRCT Findings | Text | Specify if not listed |

**HRCT FINDINGS CODING SYSTEM (8 Items)**

| **Code** | **Finding** | **Description** |
| --- | --- | --- |
| 1 | Pneumonia | Consolidation with or without ground-glass opacity indicating acute bacterial or atypical infection |
| 2 | Interstitial Lung Disease (ILD) | Includes BOOP-like patterns* |
| 3 | Pulmonary Nodule | Single or multiple nodules suggesting granulomatous disease or infection |
| 4 | Lymphadenopathy | Hilar or mediastinal lymph node enlargement |
| 5 | Abscess/ Cavity | Necrotizing lesion with cavity formation |
| 6 | Atelectasis/ Collapse | Lung volume loss from airway obstruction or compression |
| 7 | Empyema | Infected pleural collection |
| 8 | Pleural Effusion | Non-empyema fluid collection |

**Important Note on ILD/BOOP-like Pattern (Code 2):**

The two patients with ILD in this study presented with BOOP-like (bronchiolitis obliterans organizing pneumonia-like) patterns characterized by:

• Diffuse ground-glass opacities

• Mosaic attenuation on HRCT (detailed in Figure S1)

These findings reflected immune dysregulation rather than active infection, presenting with inflammatory changes mediated by dysregulated immune responses. This contrasts sharply with pneumonia-related ground-glass opacities (Code 1), which appear with consolidation and typically respond to antimicrobial therapy.

**Critical Distinction:**

• **Pneumonia (Code 1):** Acute infectious process requiring antimicrobials

**• ILD/BOOP-like pattern (Code 2):** Immune dysregulation potentially requiring immunomodulation

**• Bronchiectasis (Variable 20 in Section 4):** Chronic structural damage FROM recurrent infections (post-infectious sequela)

This pathophysiological distinction has profound therapeutic implications and is emphasized throughout the manuscript.

**Bronchoscopy and Bronchoalveolar Lavage (BAL)**

| **#** | **Variable** | **Data Type** | **Coding/ Options** |
| --- | --- | --- | --- |
| 95 | Bronchoscopy Performed | Binary | 1=Yes, 0=No |
| 96 | BAL Microbiological Results | Text | Specify organisms *(e.g., Aspergillus fumigatus, Streptococcus pneumoniae, Acinetobacter baumannii)* |

**SECTION 11: LABORATORY PARAMETERS (13 Variables)**

**Complete Blood Count**

| **#** | **Variable** | **Data Type** | **Units** | **Notes** |
| --- | --- | --- | --- | --- |
| 97 | White Blood Cell Count (WBC) | Numeric | cells/ µL (age- adjusted) | At diagnosis |
| 98 | Absolute Lymphocyte Count | Numeric | cells/ µL (age- adjusted) | At diagnosis |
| 99 | Absolute Neutrophil Count | Numeric | cells/ µL (age- adjusted) | At diagnosis |
| 100 | Hemoglobin | Numeric | g/ dL (age- adjusted) | At diagnosis |
| 101 | Platelet Count | Numeric | ×10³ cells/ µL (age- adjusted) | At diagnosis |

**Lymphocyte Subsets (Flow Cytometry)**

| **#** | **Variable** | **Data Type** | **Units** | **Reference Range** | **Notes** |
| --- | --- | --- | --- | --- | --- |
| 102 | CD3+ T cells | Numeric | cells/ µL | age- adjusted | Pre-IgRT |
| 103 | CD4+ T cells | Numeric | cells/ µL | age- adjusted | Pre-IgRT |
| 104 | CD8+ T cells | Numeric | cells/ µL | age- adjusted | Pre-IgRT |
| 105 | CD19+ B cells | Numeric | cells/ µL | age- adjusted | Pre-IgRT |

**Note on NK Cell Enumeration:**
NK (natural killer) cell enumeration was not performed as this parameter was not included in the original questionnaire and is not required by ESID/PAGID diagnostic criteria for CID, which focus primarily on T- and B-cell quantification and function.

**Serum Immunoglobulin Levels (Nephelometry)**

| **#** | **Variable** | **Data Type** | **Units** | **Reference Range** | **Notes** |
| --- | --- | --- | --- | --- | --- |
| 106 | IgG | Numeric | mg/ dL | age- adjusted | Pre-IgRT |
| 107 | IgA | Numeric | mg/ dL | age- adjusted | Pre-IgRT |
| 108 | IgM | Numeric | mg/ dL | age- adjusted | Pre-IgRT |
| 109 | IgE | Numeric | IU/ mL | age- adjusted | Pre-IgRT |

**DATA COLLECTION NOTES**

**Timing of Laboratory Assessments**

All immunological parameters (lymphocyte subsets and immunoglobulin levels, Variables 102-109) were obtained at **initial diagnostic workup prior to immunoglobulin replacement therapy (IgRT) initiation**. This timing ensures that reported values reflected inherent immunological defects rather than exogenous supplementation from therapy.

This is critical because:

- Post-IgRT immunoglobulin levels would reflect both endogenous production plus administered immunoglobulin
- Treatment-related values could mask true disease severity
- Baseline pre-therapy measurements allow accurate assessment of inherent immune dysfunction
- Comparability with published literature requires pre-treatment parameters

**Data Source**

All data were retrospectively abstracted from electronic and paper medical records by trained pediatric immunologists using this structured questionnaire. Data abstraction was performed by clinicians with specialized expertise in primary immunodeficiency disorders, ensuring accurate interpretation of complex clinical and immunological parameters.

**Age-Adjusted Interpretation**

Laboratory values were interpreted using age-adjusted reference ranges based on institutional standards derived from published pediatric references. Normal ranges vary substantially across developmental stages, particularly for lymphocyte counts and immunoglobulin levels.

**De-identification**

All patient identifiers were removed prior to analysis per institutional ethics requirements (IR.SBMU.MSP.REC.1399.692). Only de-identified data were used for research purposes.

**Data Completeness**

Not all variables were available for all patients due to the retrospective nature of data collection. Missing data were handled appropriately in statistical analyses without imputation.

**ABBREVIATIONS**

| **Abbreviation** | **Full Term** |
| --- | --- |
| AIHA | Autoimmune hemolytic anemia |
| BAL | Bronchoalveolar lavage |
| BCG | Bacille Calmette-Guérin |
| BOOP | Bronchiolitis obliterans organizing pneumonia |
| CD | Cluster of differentiation |
| CID | Combined immunodeficiency |
| ENT | Ear, nose, and throat |
| ESID | European Society for Immunodeficiencies |
| FTT | Failure to thrive |
| GI | Gastrointestinal |
| HRCT | High-resolution computed tomography |
| HSCT | Hematopoietic stem cell transplantation |
| IBD | Inflammatory bowel disease |
| Ig | Immunoglobulin |
| IgRT | Immunoglobulin replacement therapy |
| ILD | Interstitial lung disease |
| ITP | Immune thrombocytopenic purpura |
| JIA | Juvenile idiopathic arthritis |
| NK | Natural killer (cells) |
| PAGID | Pan-American Group for Immunodeficiency |
| PID | Primary immunodeficiency disease |
| WBC | White blood cell count |

**QUESTIONNAIRE ADMINISTRATION DETAILS**

**Who Completed the Questionnaire:**
Data abstraction was performed by trained pediatric immunologists with expertise in primary immunodeficiency disorders. These specialists were familiar with:

- Complex immunological laboratory interpretation
- Clinical manifestations of various primary immunodeficiency syndromes
- Nuanced differentiation between disease-related and treatment-related findings
- Age-appropriate contextualization of clinical and laboratory parameters

**Data Sources:**

- Electronic medical records
- Paper medical records
- Laboratory information systems
- Radiology information systems
- Pharmacy records (for treatment documentation)

**Quality Control:**

- Double data entry for critical variables
- Regular team meetings to resolve ambiguous cases
- Consultation with treating physicians for clarification when needed

**SPECIAL NOTES ON KEY VARIABLES**

**Bronchiectasis (Variable 20)**

Bronchiectasis in this study represents chronic irreversible structural damage resulting from recurrent bacterial infections. It is coded as present based on:

- HRCT findings of bronchial wall thickening and bronchial dilation
- Clinical history of recurrent productive cough
- Evidence of chronic bacterial colonization

This is distinct from traction bronchiectasis associated with fibrotic lung disease and represents post-infectious sequelae requiring antimicrobial-focused management.

**Interstitial Lung Disease/BOOP-like Pattern (HRCT Code 2)**

Two patients exhibited ILD with BOOP-like patterns characterized by:

- Diffuse ground-glass opacities
- Mosaic attenuation
- Absence of consolidation suggesting bacterial pneumonia

These findings suggested immune dysregulation as the primary pathogenic mechanism rather than active infection. Representative imaging is provided in Figure S1.

**Pre-IgRT Timing (Variables 106-109)**

Immunoglobulin levels were specifically obtained before initiation of immunoglobulin replacement therapy to ensure values reflected endogenous production. This is explicitly noted in the data collection protocol and verified through review of treatment records.

**CONTACT INFORMATION**

For questions regarding this questionnaire or data collection methodology:

**Dr. Zahra Chavoshzadeh**
Corresponding Author
Mofid Children's Hospital
Shahid Beheshti University of Medical Sciences
Tehran, Iran
Email: zahra_chavoshzadeh@yahoo.com
Phone: +98-21-22907517
