# Supplementary material for: Demographic, clinical, and immunological features in combined immunodeficiency patients: a comparative analysis of those with and without pulmonary manifestations – a multicenter study from Iran
Source: BMC Pulm Med. 2026 Jan 31;26:100. doi: 10.1186/s12890-026-04115-3 (PMC12947537; doi:10.1186/s12890-026-04115-3)
Supplement: Supplementary file 2 — Supplementary Material 2. Figure S1.1 - HRCT demonstrating an extensive BOOP-like pattern in a 2-year-old male CID patient, characterized by diffuse bilateral ground-glass opacities and mosaic attenuation, illustrating immune-mediated interstitial lung disease. Figure S1.2 - HRCT demonstrating a BOOP-like pattern in a 5-year-old male CID patient, showing bilateral ground-glass opacities with mosaic attenuation, characteristic of immune dysregulation-mediated lung injury. Figure S1.3 - HRCT in a 12-year-old male with CID demonstrating cylindrical bronchiectasis, bronchial wall thickening, and a 'tree-in-bud' pattern, representing chronic infectious sequelae of recurrent pneumonia. [file 12890_2026_4115_MOESM2_ESM.docx]

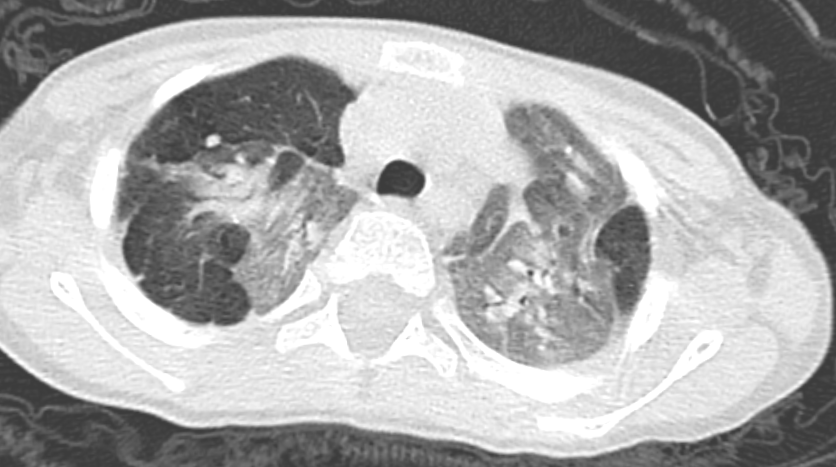


**Figure S1.1** - HRCT demonstrating an extensive BOOP-like pattern in a 2-year-old male CID patient, characterized by diffuse bilateral ground-glass opacities and mosaic attenuation, illustrating immune-mediated interstitial lung disease.


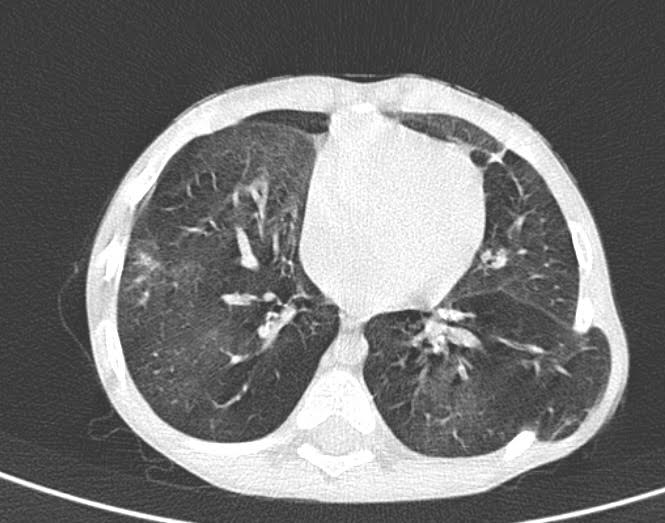


**Figure S1.2** - HRCT demonstrating a BOOP-like pattern in a 5-year-old male CID patient, showing bilateral ground-glass opacities with mosaic attenuation, characteristic of immune dysregulation-mediated lung injury.


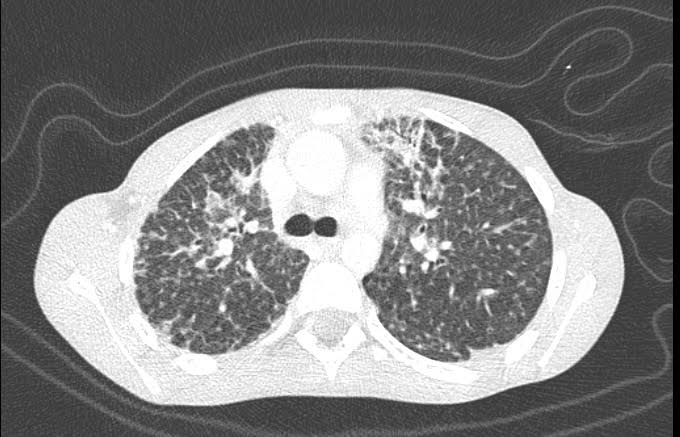


**Figure S1.3** - HRCT in a 12-year-old male with CID demonstrating cylindrical bronchiectasis, bronchial wall thickening, and a 'tree-in-bud' pattern, representing chronic infectious sequelae of recurrent pneumonia.
